# Efficacy and Safety of Ceftaroline for the Treatment of Community-Acquired Pneumonia: A Systemic Review and Meta-Analysis of Randomized Controlled Trials

**DOI:** 10.3390/jcm8060824

**Published:** 2019-06-09

**Authors:** Shao-Huan Lan, Shen-Peng Chang, Chih-Cheng Lai, Li-Chin Lu, Chien-Ming Chao

**Affiliations:** 1 School of Pharmaceutical Sciences and Medical Technology, Putian University, Putian 351100, Fujian, China; shawnlan0713@gmail.com; 2 Department of Pharmacy, Chi Mei Medical Center, Liouying 73657, Taiwan; httremoon@ms.szmc.edu.tw; 3 Department of Intensive Care Medicine, Chi Mei Medical Center, Liouying 73657, Taiwan; dtmed141@gmail.com; 4 School of Management, Putian University, Putian 351100, Fujian, China; jane90467@gmail.com

**Keywords:** ceftaroline, ceftriaxone, community-acquired pneumonia, safety

## Abstract

This study aimed to compare the clinical efficacy and safety of ceftaroline with those of ceftriaxone for treating community-acquired pneumonia (CAP). The PubMed, Cochrane Library, Embase, and clinicalTrials.gov databases were searched until April 2019. This meta-analysis only included randomized controlled trials (RCTs) that evaluated ceftaroline and ceftriaxone for the treatment of CAP. The primary outcome was the clinical cure rate, and the secondary outcome was the risk of adverse events (AEs). Five RCTs were included. Overall, at the test of cure (TOC), the clinical cure rate of ceftaroline was superior to the rates of ceftriaxone for the treatment of CAP (modified intent-to-treat population (MITT) population, odds ratio (OR) 1.61, 95% confidence interval (CI) 1.31–1.99, *I*^2^ = 0%; clinically evaluable (CE) population, OR 1.38, 95% CI 1.07–1.78, *I*^2^ = 14%). Similarly, the clinical cure rate of ceftaroline was superior to that of ceftriaxone at the end of therapy (EOT) (MITT population, OR 1.57, 95% CI 1.16–2.11, *I*^2^ = 0%; CE population, OR 1.64, 95% CI 1.15–2.33, *I*^2^ = 0%). For adult patients, the clinical cure rate of ceftaroline remained superior to that of ceftriaxone at TOC (MITT population, OR 1.66, 95% CI 1.34–2.06, *I*^2^ = 0%; CE population, OR 1.39, 95% CI 1.08–1.80, *I*^2^ = 30%) and at EOT (MITT population, OR 1.64, 95% CI 1.20–2.24, *I*^2^ = 0%; CE population, OR 1.65, 95% CI 1.15–2.36, *I*^2^ = 0%). Ceftaroline and ceftriaxone did not differ significantly in the risk of serious AEs, treatment-emergent AEs, and discontinuation of the study drug owing to an AE. In conclusion, the clinical efficacy of ceftaroline is similar to that of ceftriaxone for the treatment of CAP. Furthermore, this antibiotic is as tolerable as ceftriaxone.

## 1. Introduction

Community-acquired pneumonia (CAP) is a common acute bacterial infection among adults and children and has become a significant global health problem [1,2,3,4]. Moreover, severe CAP is associated with high morbidity and mortality, particularly when prompt and appropriate treatment is not provided [5,6]. However, the emergence of antibiotic resistance in this era—with the increase in resistant bacteria not treatable with existing antibiotics—and the lack of development of novel antibiotics has complicated the use of antibiotics unlike before [3,7]. In addition to the most common CAP pathogen—*Streptococcus pneumoniae*, less than 8% of CAP can be caused by the so-called PES pathogens—*Pseudomonas aeruginosa*, extended-spectrum β-lactamase producing *Enterobacteriaceae*, and methicillin-resistant *Staphylococcus aureus* (MRSA), especially in intensive care unit (ICU) [8,9]. Among PES, MRSA is the most frequently reported, and it requires the use of specific antimicrobial agents for the treatment of typical CAP [10]. Currently, the antibiotics recommended for treating CAP when MRSA infection is suspected are vancomycin, teicoplanin, and linezolid [11,12,13].

Ceftaroline is a new cephalosporin with broad-spectrum activity against many commonly encountered pathogens causing CAP, including *S. pneumoniae, S. aureus, Moraxella catarrhalis, Haemophilus influenzae, and Klebsiella pneumonia* [14,15,16]. Moreover, several investigations have demonstrated the substantial in vitro activity of ceftaroline against MRSA from various clinical specimens, including skin/soft tissue and respiratory tract [15,17,18,19]. Global surveillance revealed that compared to ceftriaxone, ceftaroline showed superior in vitro activity against common CAP pathogens [17]. Subsequently, several randomized controlled trials (RCTs) [20,21,22,23,24] have investigated the efficacy and safety of ceftaroline for the treatment of CAP. In the present study, we conducted a comprehensive meta-analysis to provide high-quality evidence on the efficacy and safety of ceftaroline compared to those of ceftriaxone for treating CAP.

## 2. Methods

### 2.1. Study Search and Selection

All clinical studies were identified through a systematic review of the literature in the PubMed, Embase, ClinicalTrials.gov, and Cochrane databases until April 2019 using the following search terms: “ceftaroline”, “Teflaro”, “Zinforo”, “pneumonia”, and “RCT”. Only RCTs that compared the clinical efficacy and adverse effects of ceftaroline and ceftriaxone were included. Two reviewers (Lan and Chang) searched and examined publications independently to avoid bias. When they disagreed, a third author (Lai) resolved the issue. The following data were extracted from each study included in the meta-analysis: year of publication, study design, duration, antibiotic regimens of ceftaroline and ceftriaxone, outcomes, and adverse events (AEs).

### 2.2. Definitions and Outcomes

The primary outcome was the overall clinical cure with the resolution of clinical signs and symptoms of pneumonia or improvement to the extent that no further antimicrobial therapy was necessary at the end of therapy (EOT) and test of cure (TOC) in the modified intent-to-treat population (MITT) and the clinically evaluable (CE) population. The EOT visit took place within 48 h after the last dose of oral study drug or within 24 h after the last dose of the IV study drug. The TOC visit was at 8–15 days after the last dose of the IV or oral study drug (whichever was given last). Patients in the MITT population who met minimal disease criteria and had ≥1 bacterial pathogen commonly associated with CAP identified at baseline were included in the microbiological modified MITT (mMITT) population, and those who met criteria for both the CE and mMITT populations were included in the microbiologically evaluable (ME) population. The secondary outcome was the risk of AEs, including mild, moderate, and severe degree and discontinuation because of AEs, relapse rate, and mortality.

### 2.3. Data Analysis

This study used the Cochrane risk-of-bias tool to assess the quality of enrolled RCTs and their risk of bias [25]. The Review Manager software program, version 5.3, was used to conduct statistical analyses. The degree of heterogeneity was evaluated using the Q statistic generated from the χ^2^ test. The *I*^2^ measure assessed the proportion of statistical heterogeneity. Heterogeneity was considered significant when the *P* value was less than 0.10 or the *I*^2^ value was more than 50%. The random-effects model was used when data were significantly heterogeneous, and the fixed-effect model was used when the data were homogeneous. Pooled odds ratios (ORs) and 95% confidence intervals (CI) were calculated for outcome analyses.

## 3. Results

**Figure 1:**
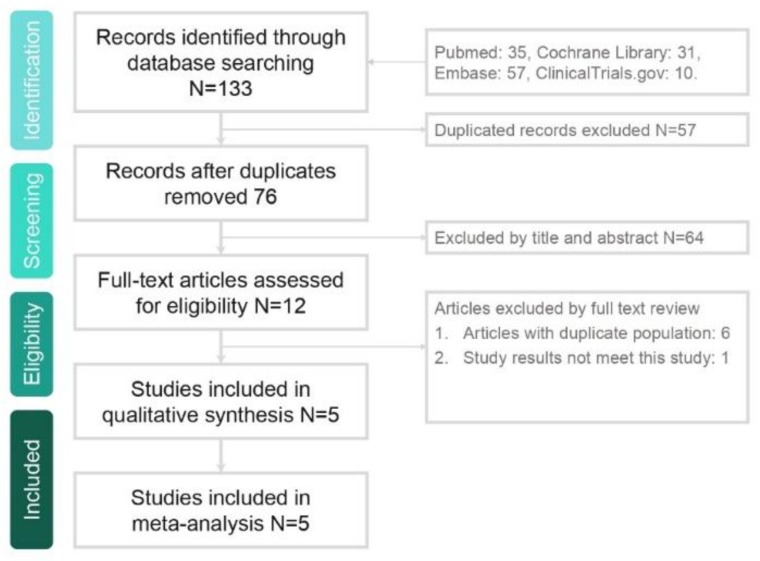
Flowchart of the study selection process.

The search results yielded a total of 133 studies from the online databases, and 76 studies were excluded because of duplication. Additionally, 64 studies were found to be irrelevant after the title and abstract were screened (article type and language), and 7 studies were found to be irrelevant after the full text was screened. Eventually, five RCTs [20,21,22,23,24] were enrolled for the meta-analysis (Figure 1).

### 3.1. Study Characteristics and Study Quality

**Figure 2:**
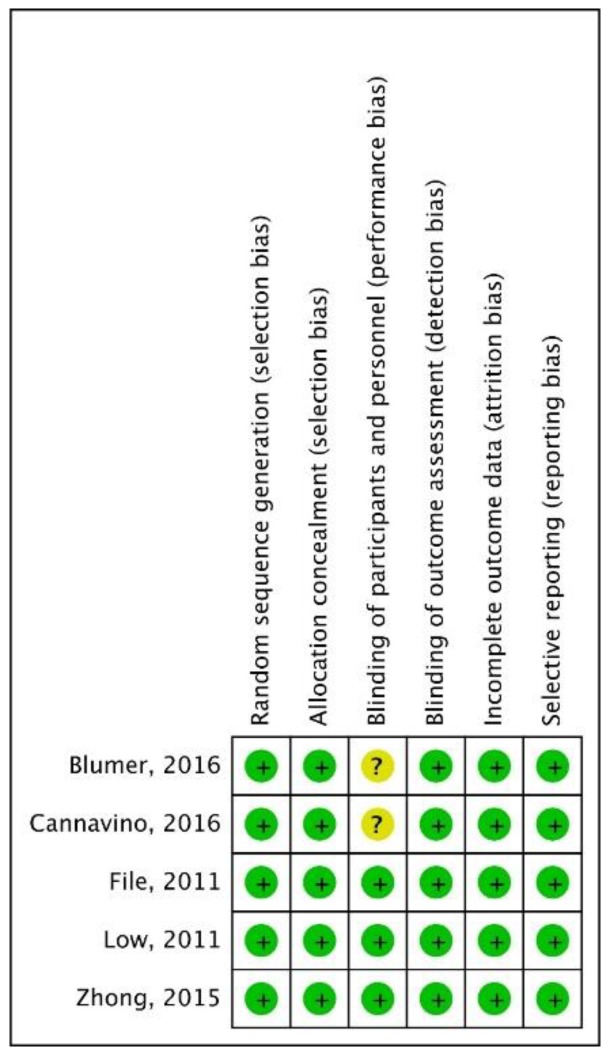
Risk of bias per study and domain.

All five RCTs [20,21,22,23,24] included were multinational and multicenter studies (Table 1). Three studies [20,21,22] focused on adult patients with CAP with Pneumonia Outcomes Research Term (PORT) [26] risk class III–IV, and two studies [23,24] enrolled pediatric patients only. Overall, the experimental group treated with ceftaroline and the control group treated with ceftriaxone comprised 1153 and 1050 patients, respectively. Almost all risks of basis in each study were low (Figure 2).

### 3.2. Clinical Efficacy

**Figure 3:**
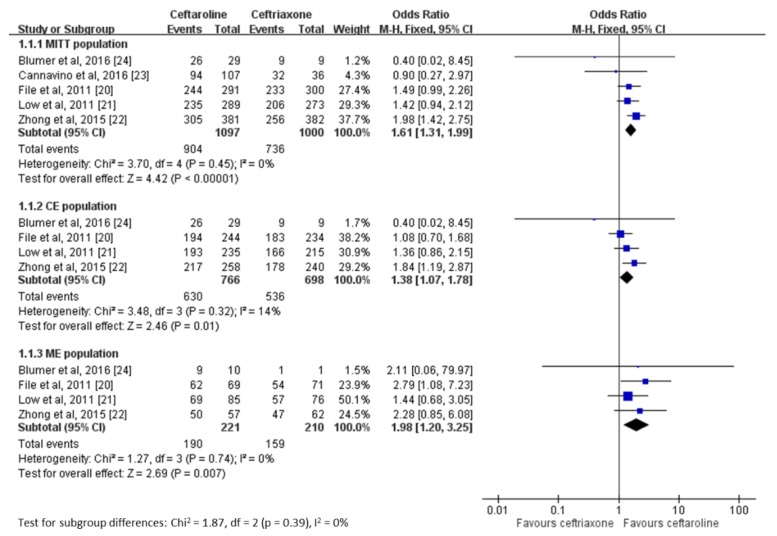
Overall clinical cure rates of ceftaroline and ceftriaxone for the treatment of community-acquired pneumonia. MITT, modified intent-to-treat population; CE, clinically evaluable; ME, microbiologically evaluable.

Notably, ceftaroline had a superior clinical cure rate at TOC compared with ceftriaxone for the treatment of CAP (MITT population, OR 1.61, 95% CI 1.31–1.99, *I*^2^ = 0%; CE population, OR 1.38, 95% CI 1.07–1.78, *I*^2^ = 14%; ME population, OR 1.98, 95% CI 1.20–3.25, *I*^2^ = 0%; Figure 3). Similarly, at EOT, the clinical cure rate of ceftaroline was superior compared with that of ceftriaxone (MITT population, OR 1.57, 95% CI 1.16–2.11, *I*^2^ = 0%; CE population, OR 1.64, 95% CI 1.15–2.33, *I*^2^ = 0%).

In the subgroup analysis of three studies [20,21,22] including adult patients, the clinical cure rate of ceftaroline remained superior to that of ceftriaxone at TOC (MITT population, OR 1.66, 95% CI 1.34–2.06, *I*^2^ = 0%; CE population, OR 1.39, 95% CI 1.08–1.80, *I*^2^ =30%) and at EOT (MITT population, OR 1.64, 95% CI 1.20–2.24, *I*^2^ = 0%; CE population, OR 1.65, 95% CI 1.15–2.36, *I*^2^ =0%). On the other hand, the pooled analysis of two studies showed that the clinical cure rates at TOC and EOT were similar between pediatric patients treated with ceftaroline or ceftriaxone (at TOC, OR 0.79, 95% CI 0.26–2.97, *I*^2^ = 0%; at EOT, OR 1.02, 95% CI 0.38–2.75, *I*^2^ = 0%)[23,24].

**Figure 4:**
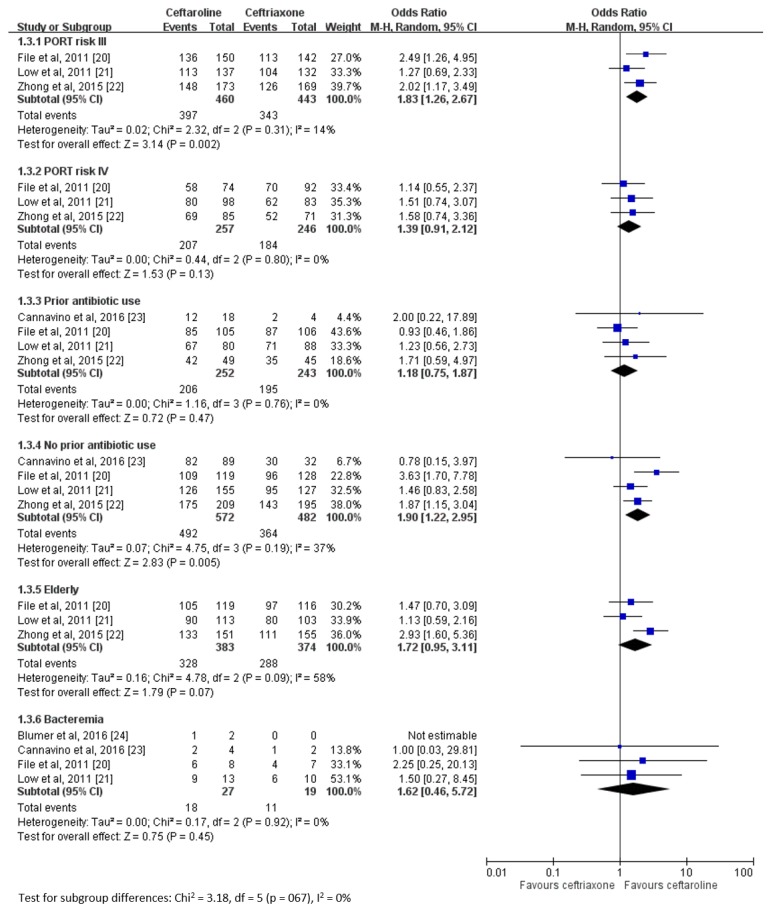
Overall clinical cure rates of ceftaroline and ceftriaxone for the treatment of community-acquired pneumonia based on patient group.

Figure 4 shows further analysis of the clinical cure rate (ceftaroline vs. ceftriaxone) at the TOC visit in various patient subgroups. Ceftaroline showed a superior clinical cure rate than ceftriaxone for patients with PORT risk III (OR 1.83, 95% CI 1.26–2.67, *I*^2^ = 14%) but not for patients with PORT risk IV (OR 1.39, 95% CI 0.91–2.12, *I*^2^ = 0%). The efficacy of ceftaroline was superior compared to that of ceftriaxone in patients who did not receive prior antibiotics (OR 1.90, 95% CI 1.22–2.95, *I*^2^ = 37%) but not in those who received prior antibiotics (OR 1.18, 95% CI 0.75–1.87, *I*^2^ = 0%). No differences were observed in the clinical cure rate between elderly patients (age ≥65 years) treated with ceftaroline or ceftriaxone (OR 1.72, 95% CI 0.95–3.11, *I*^2^ = 58%) and between patients with bacteremia treated with ceftaroline or ceftriaxone (OR 1.62, 95% CI 0.46–5.72, *I*^2^ = 0%).

**Figure 5:**
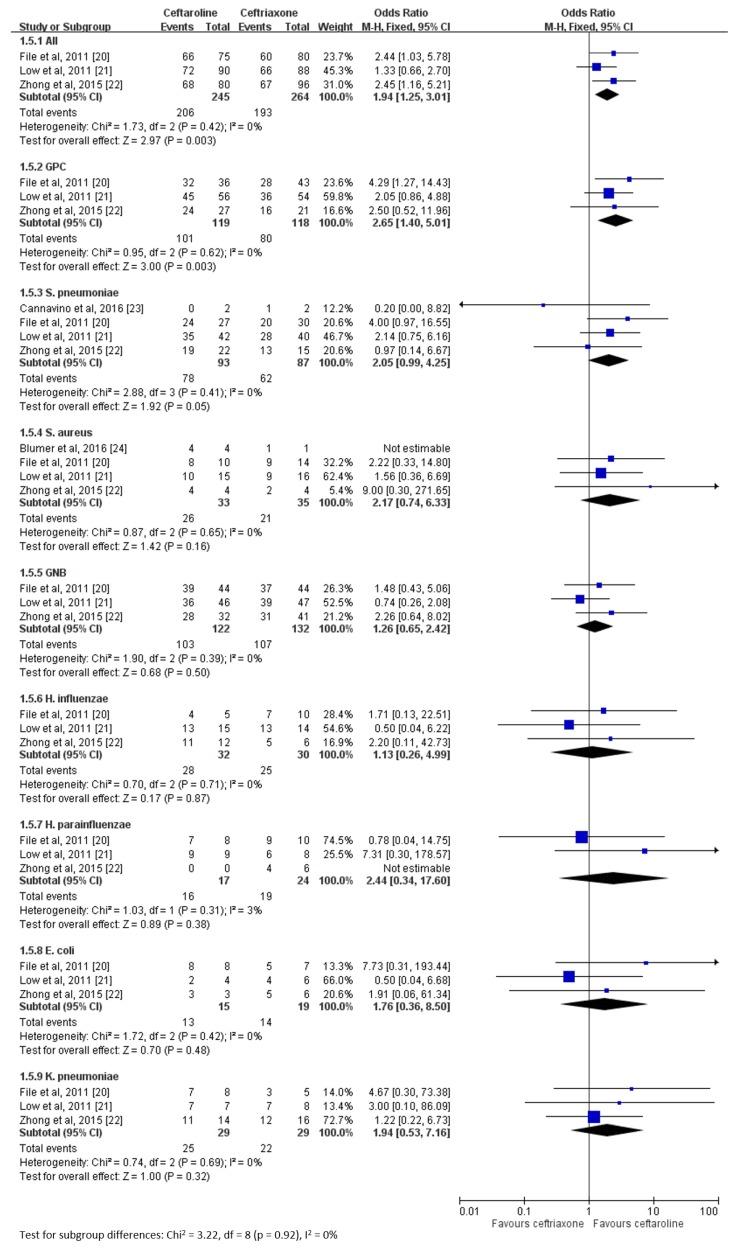
Overall clinical cure rates of ceftaroline and ceftriaxone for the treatment of community-acquired pneumonia based on pathogens.

We also assessed the clinical cure rate based on pathogens among the mMITT population, and we found that ceftaroline was superior to ceftriaxone in the overall population (OR 1.94, 95% CI 1.25–3.01, *I*^2^ = 0%, Figure 5). Ceftaroline was superior to ceftriaxone in patients with gram-positive coccal (GPC) infection (OR 2.65, 95% CI 1.40–5.01, *I*^2^ = 0%) but not in those with gram-negative bacterial (GNB) infection (OR 1.26, 95% CI 0.65–2.42, *I*^2^ = 0%). No significant difference was noted between the ceftaroline and ceftriaxone groups for each of the following pathogens: *S. pneumoniae, S. aureus, H. influenzae, H. parainfluenzae, Escherichia coli,* and *K. pneumoniae*.

### 3.3. Adverse Events

**Figure 6:**
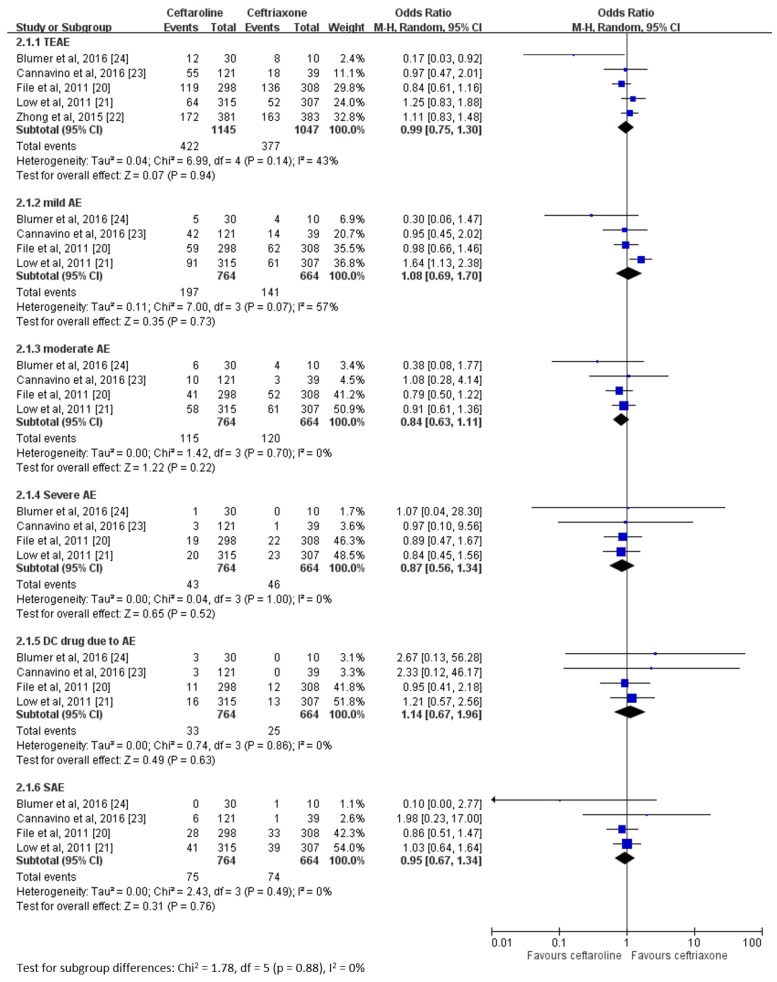
Risk of adverse events between ceftaroline and ceftriaxone for the treatment of community-acquired pneumonia.

No significant differences were observed in the risk of overall treatment-emergent adverse events (TEAEs) between the ceftaroline and ceftriaxone groups (OR 0.99, 95% CI 0.75–1.30, *I*^2^ = 43%), and the similarity was not changed by the degree of severity (Figure 6). The risks of serious AEs and discontinuation of the study drug were similar between the ceftaroline and ceftriaxone groups (Figure 5). In addition, no relapse was noted among all enrolled patients. Finally, the mortality rate was similar between the ceftaroline and ceftriaxone groups (OR 1.13, 95% CI 0.57–2.23, *I*^2^ = 0), and none of the cases of mortality were related to the study drug.

## 4. Discussion

This meta-analysis of five RCTs determined that the clinical efficacy of ceftaroline was superior to that of ceftriaxone for the treatment of patients with CAP. First, the overall clinical cure rate of ceftaroline was superior to that of ceftriaxone for treating CAP in the pooled populations of the five RCTs, including pediatric and adult patients [20,21,22,23,24]. The superiority of ceftaroline compared to ceftriaxone remained significant at different times of outcome measurement, including EOT and TOC, and in different populations, including MITT, CE, and ME populations. Second, we found that ceftaroline had a higher clinical cure rate than ceftriaxone among adult patients in the subgroup analysis of three studies [20,21,22] including adult patients, but the pooled analysis of two studies [23,24] including pediatric patients showed similar clinical cure rates for ceftaroline and ceftriaxone. However, the two studies [23,24] that focused on pediatric patients had a limited number of patients. Therefore, more pediatric studies are warranted to clarify this issue. Third, the subgroup analysis of CAP in various populations demonstrated that ceftaroline was at least similar to ceftriaxone in patients with PORT risk IV, those who received previous antibiotics, those who were aged ≥65 years, and those with bacteremia but superior to ceftriaxone in patients with PORT risk III and those who did not receive previous antibiotics. In summary, the overall clinical efficacy of ceftaroline is similar to that of ceftriaxone for the treatment of CAP. For other populations, ceftaroline is at least similar to ceftriaxone in terms of the clinical cure rate. However, the case numbers of several subgroup analyses, such as bacteremia, PORT risk IV or different pathogens were limited, which may limit the significance of differences between ceftaroline and ceftriaxone. Therefore, a further large-scale study is warranted to prove our findings.

In the mMITT population, the present meta-analysis determined that the clinical cure rate of ceftaroline was superior to that of ceftriaxone for CAP caused by GPC, but no significant difference was found for CAP caused by GNB, *S. pneumoniae, S. aureus, H. influenzae, H. parainfluenzae, E. coli,* and *K. pneumoniae* between the ceftaroline and ceftriaxone groups. The effectiveness of ceftaroline for the treatment of CAP is supported by in vitro studies. In a surveillance study at a US medical center, ceftaroline was noted to be more potent against *S. pneumoniae* (MIC_50_ ≤ 0.015 vs. ≤ 0.06 μg/mL; MIC_90_ = 0.12 vs. 1 μg/mL) and even remained active against strains nonsusceptible to ceftriaxone (MIC_90_ = 0.25 μg/mL) [18]. Similar findings were demonstrated in the analysis of bacterial isolates in pediatric patients [15]. Upon global surveillance, ceftaroline was noted to be more potent than ceftriaxone against MSSA and *S. pneumoniae,* and ceftaroline had similar efficacy to ceftriaxone against *H. influenzae* [17]. Overall, the in vitro activity of ceftaroline that is greater or at least equal to ceftriaxone against most commonly encountered pathogens causing CAP could largely explain the high in vivo clinical response in this meta-analysis. However, we can only see the trend of better efficacy of ceftaroline than the comparator in each subgroup; these differences do not reach statistical significance. This may be due to the limited case number of each pathogen, so further large-scale study is warranted.

Although this study demonstrated the clinical efficacy of ceftaroline in the treatment of CAP, antibiotics may have a limited effect on the outcome of CAP, particularly these severely affected cases. This could be due to the fact that pneumonia is caused by a variety of pathogens, including respiratory viruses, Mycoplasma pneumoniae, and bacteria. In addition, incidence of primary bacterial pneumonia may be very low and be far less than that of nonbacterial pneumonia in developed countries as well as in developing countries [27]. Incidences of each pathogen pneumonia may differ in children and adults (older persons) across the populations, but severe pneumonia of viral or nonpathogen origin can induce secondary bacterial infection caused by lung injuries from primary insults; hence, it is reasonable that any pneumonia patients could be treated with antibiotics. However, antibiotics have a limited effect on the natural course of infection-related extrapulmonary manifestations. Further, outcomes of severe pneumonia may be affected by underlying comorbidities or the immune status of the host, not only by antibiotic treatment. Moreover, the pattern of antimicrobial resistance may vary in different sites; therefore, the guidelines for antibiotic treatment for CAP may differ and could be changed in each country over time. In summary, although the appropriate use of antibiotics is essential for the successful treatment of pneumonia, many factors, including disease severity, underlying comorbidity, immune status, pathogens, and the timing of antibiotic use are also significantly associated with the outcome of pneumonia.

The risk of AEs is another major concern when treating CAP with this antimicrobial agent. The most common AEs are headache, diarrhea, and insomnia [28]. In this analysis, the pooled risks of TEAEs of all degrees and even serious AEs were similar between the ceftaroline and ceftriaxone groups. Additionally, ceftaroline is associated with the risk of discontinuation of the study drug that is similar to that of ceftriaxone; this risk is because of the development of AEs. Although the overall mortality of the ceftaroline group was only 1.81%—which was comparable to that of ceftriaxone group—none of the cases of mortality were associated with the study drug. Therefore, all these findings revealed that ceftaroline is as safe as ceftriaxone for the treatment of CAPs.

A major strength of this meta-analysis is that only RCTs were included, thereby reducing the risk of bias and providing strong evidence. However, this meta-analysis also has several limitations. First, the number of MRSA-associated pneumonia cases was limited in this study. Therefore, the anti-MRSA effect of ceftaroline, which is not owned by ceftriaxone, cannot be elucidated in this meta-analysis. Second, this meta-analysis had a limited number of studies and patients in subgroup analyses, such as different pathogens among different age groups. Therefore, some differences between the ceftaroline and ceftriaxone groups did not reach statistical significance.

## 5. Conclusions

In conclusion, based on the findings of this meta-analysis of five RCTs, the clinical efficacy of ceftaroline is similar to that of ceftriaxone for the treatment of CAP. Additionally, ceftaroline was as tolerable as ceftriaxone. However, clinicians should cautiously use ceftaroline in the selected population at high risk of MRSA to avoid the unnecessary coverage of MRSA by ceftaroline. Overall, ceftaroline can be recommended as an appropriate antibiotic therapy for CAP.

## Figures and Tables

**Table 1:** Characteristics of included studies.

Study, Published Year	Study Design	Study Period	Study Population	No of Patients		Dose Regimen	
				Ceftaroline	Comparator	Ceftaroline	Comparator
File et al., 2011 [20]	Multicenter, multinational, double-blinded, randomized trial	January 2008 to December 2008	Adult patients with PORT risk class III or IV CAP requiring hospitalization and IV therapy	304	309	600 mg q12 h	Ceftriaxone 1 g q24 h
Low et al., 2011 [21]	Multicenter, multinational, double-blinded, randomized trial	2007–2009	Patients (aged ≥18 years) with PORT risk class III or IV CAP requiring hospitalization and IV therapy	317	310	600 mg q12 h	Ceftriaxone 1 g q24 h
Zhong et al., 2015 [22]	Multicenter, multinational, double-blinded, randomized trial	2011–2013	Adult Asian patients with PORT risk class III–IV CAP	381	382	600 mg q12 h	Ceftriaxone 2 g q24 h
Cannavino et al., 2016 [23]	Multicenter, multinational, randomized	2012–2014	Ages of 2 months and <18 years with CAP requiring hospitalization and IV antibacterial therapy	121	39	Age < 6 m, 8 mg/kg q8 h; aged ≥ 6 m, 12 mg/kg q8 h for those weighing ≤ 33 kg or 400 mg q8 h for those weighing >33 kg	Ceftriaxone 75 mg/kg/d to a maximum 4g/d q12 h
Blumer et al., 2016 [24]	Multicenter, multinational randomized, observe-blinded	2012–2014	Pediatric patients between 2 months and 17 years of age with complicated CAP	30	10	15 mg/kg or 600 mg q8 h if weight > 40 kg if ≥6 m or 10 mg/kg q8 h if <6 m	Ceftriaxone, 75 mg/kg/d q12 h, and vancomycin 15 mg/kg q6 h
